# Long-Read 16S rRNA Amplicon and Metagenomic Data of Swine Feed-Additive Probiotics Product

**DOI:** 10.1128/mra.00397-22

**Published:** 2022-08-22

**Authors:** Aunthikarn Sudjai, Kantgamon Suriye, Worarat Kruasuwan, Piroon Jenjaroenpun, Suporn Foongladda, Thidathip Wongsurawat

**Affiliations:** a Division of Bioinformatics and Data Management for Research, Research Group and Research Network Division, Research Department, Faculty of Medicine Siriraj Hospital, Mahidol University, Bangkok, Thailand; b Siriraj Long-Read Lab (Si-LoL), Faculty of Medicine Siriraj Hospital, Mahidol University, Bangkok, Thailand; c Department of Microbiology, Faculty of Medicine Siriraj Hospital, Mahidol University, Bangkok, Thailand; Loyola University Chicago

## Abstract

Swine feed-additive probiotics products play a major role in swine performance and welfare by promoting gut health. Here, we present two types of data, including a full-length 16S rRNA amplicon sequence data and a long-read metagenomic sequence data obtained from the same commercial probiotic product.

## ANNOUNCEMENT

Supplementing beneficial microorganisms to reduce the use of antibiotics and enhance the growth of animals is an ultimate goal of sustainable livestock production (1). In spite of the promotion of animal protein demand, more than half of all antibiotics are still used in the agricultural industry as feed additives, especially in the swine farming farm industry (2, 3). Despite the fact that the product has specific information on a probiotic product label regarding microorganism names and statement of quantity (4), the microbiological composition of several commercial probiotic products does not correspond to the product label (5, 6). In this work, both 16S-rRNA gene amplification and metagenomic (amplification-free) method were applied to identify species in a probiotic product.

Swine feed-additive probiotics product available in Thailand was collected from a market in order to try two different nanopore library preparation protocols, i.e., a full-length 16S rRNA amplicon and metagenomic sequencing approaches. To obtain the bacterial pellet, 15 mL of liquid probiotics product was centrifuged for 15 min at 4,500 rpm. The pellet was used for genomic DNA extraction by ZymoBIOMICS DNA miniprep kit protocol (D4300, Zymo Research, USA). The full-length 16S amplicon was amplified using LongAmp *Taq* 2× Master Mix (New England Biolabs, UK) with the following conditions: 95°C for 1 min, 25 cycles of 95°C for 20 s, 55°C for 30 s, and 65°C for 2 min, followed by 65°C for 5 min, and prepared the library using the 16S Barcoding kit (SQK-RAB204) protocol (ONT, UK). For metagenomic sequencing, the library preparation was performed using the Rapid Barcoding kit (RBK004, ONT, UK). Briefly, the metagenomic DNA used for the library preparation was cleaved with transposase enzyme to produce chemically modified ends, and a barcode was added to each DNA sample, finally ligated with an adapter. MinION (Mk1C) sequencing was performed using an R9.4.1 flow cell (version: FLO-MIN106; ONT) with the default setting. Base calling and demultiplexing were performed using Guppy version 6.0.1 in the SUP (super accuracy) mode (7). Adapters and barcodes were removed from the reads using Porechop version 0.2.4 (https://github.com/rrwick/Porechop). Read qualities were assessed with Nanoplot version 1.20.0 (7). The reads, which have a mean quality score of >10 with at least 1000-bp read length for 16S amplicon and >9 with at least 200-bp for shotgun sequencing, were kept using NanoFilt version 2.8.0 (7) for the next step.

**FIG 1:**
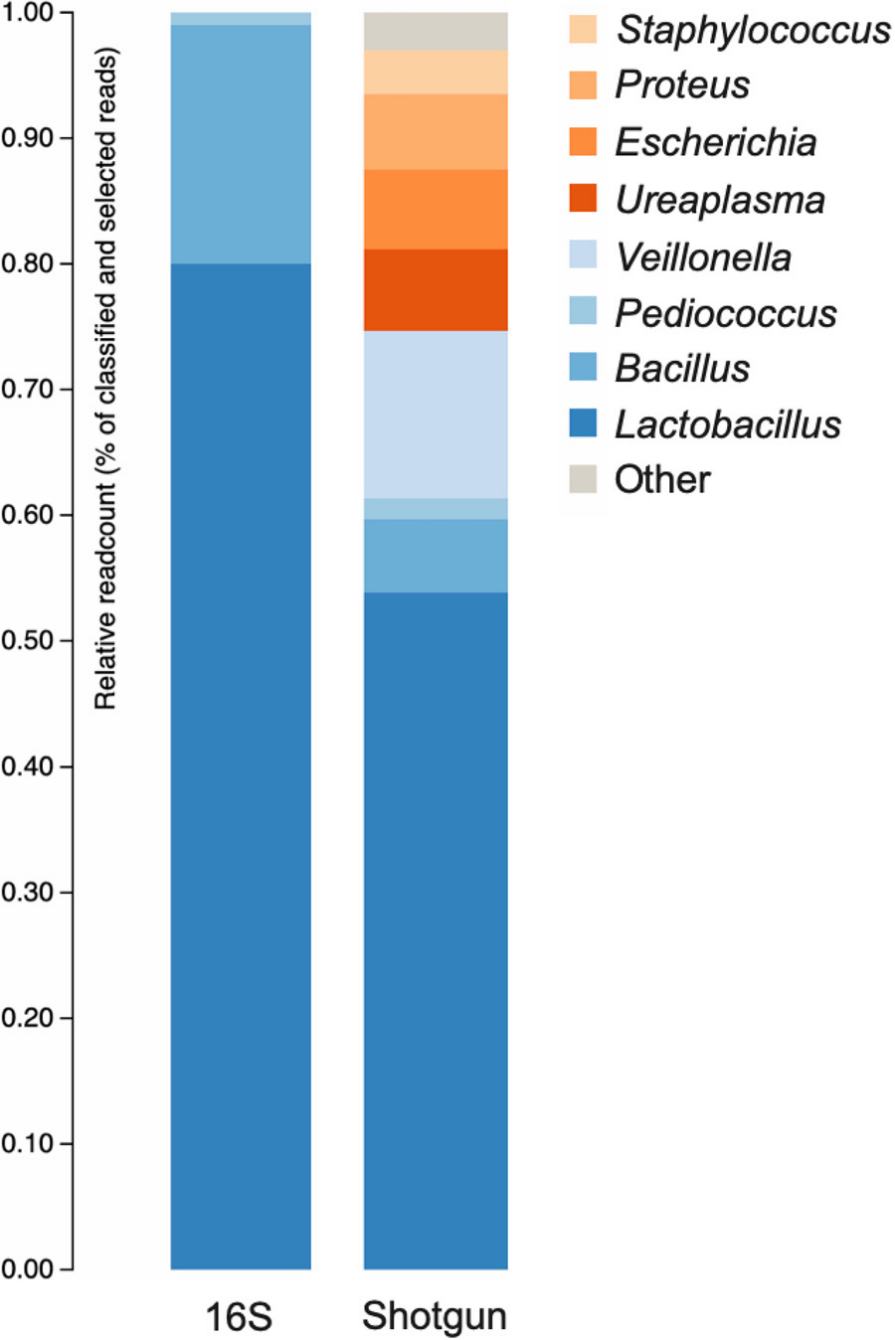
Relative abundance plot of the microbial communities in the swine feed-additive probiotics product based on 16S rRNA gene amplicon and shotgun sequencing. Each bar represents the relative frequency of each microbial genera.

Taxonomic classification was performed using the One Codex platform (https://app.onecodex.com). Full-length 16S amplicon sequencing yielded a total of 143,044 DNA sequences with 1,109,395,320 bases and a mean read length of 1469-bp. While metagenome analysis yielded a total of 504,161 reads with 468,747,357 bases and a read length *N*_50_ value of 3,435-bp. Both 16S amplicon and metagenomic sequences were taxonomically annotated against the NCBI RefSeq targeted loci database, which include protein-coding or rRNA loci such as 16S rRNA genes, 18S rRNA genes, 28S rRNA genes gene, and internal transcribed spacer (ITS) (8). Our result revealed that the genera *Lactobacillus*, *Bacillus*, and *Pediococcus* were dominant in both 16S amplicon and shotgun metagenomic data. Nonetheless, *Veillonella*, *Ureaplasma*, Escherichia, Proteus, and Staphylococcus genera were only classified in the metagenomic data (Fig. 1). This result demonstrated that metagenomic data outperforms 16S amplicon data in microbial identification. Targeted 16S rRNA gene sequencing through amplification may introduce PCR biases in bacterial quantifying taxa, resulting in underestimating of bacterial genera abundance, as investigated in previously reports (9, 10) and this study.

### Data availability.

The raw sequencing data are available at the NCBI Sequence Read Archive (SRA) under BioProject PRJNA823500 with accession numbers SRX14783968 (16S amplicon) and SRX14783970 (metagenomic sequencing).

## References

[1] Hosain MZ, Kabir SML, Kamal MM. 2021. Antimicrobial uses for livestock production in developing countries. Vet World 14:210–221. doi:10.14202/vetworld.2021.210-221. 33642806 PMC7896880

[2] Lekagul A, Tangcharoensathien V, Liverani M, Mills A, Rushton J, Yeung S. 2021. Understanding antibiotic use for pig farming in Thailand: a qualitative study. Antimicrob Resist Infect Control 10:3. doi:10.1186/s13756-020-00865-9. 33407887 PMC7789695

[3] Chan R, Chiemchaisri C, Chiemchaisri W, Boonsoongnern A, Tulayakul P. 2022. Occurrence of antibiotics in typical pig farming and its wastewater treatment in Thailand. Emerg Contam 8:21–29. doi:10.1016/j.emcon.2021.12.003.

[4] Jackson SA, Schoeni JL, Vegge C, Pane M, Stahl B, Bradley M, Goldman VS, Burguière P, Atwater JB, Sanders ME. 2019. Improving end-user trust in the quality of commercial probiotic products. Front Microbiol 10:739. doi:10.3389/fmicb.2019.00739. 31105649 PMC6499161

[5] Huys G, Vancanneyt M, D'Haene K, Vankerckhoven V, Goossens H, Swings J. 2006. Accuracy of species identity of commercial bacterial cultures intended for probiotic or nutritional use. Res Microbiol 157:803–810. doi:10.1016/j.resmic.2006.06.006. 16919915

[6] Wang Y, Liang Q, Lu B, Shen H, Liu S, Shi Y, Leptihn S, Li H, Wei J, Liu C, Xiao H, Zheng X, Liu C, Chen H. 2021. Whole-genome analysis of probiotic product isolates reveals the presence of genes related to antimicrobial resistance, virulence factors, and toxic metabolites, posing potential health risks. BMC Genom 22:210. doi:10.1186/s12864-021-07539-9. PMC7988973 33761872

[7] De Coster W, D'Hert S, Schultz DT, Cruts M, Van Broeckhoven C. 2018. NanoPack: visualizing and processing long-read sequencing data. Bioinformatics 34:2666–2669. doi:10.1093/bioinformatics/bty149. 29547981 PMC6061794

[8] Minot SS, Krumm N, Greenfield NB. 2015. One Codex: a sensitive and accurate data platform for genomic microbial identification. bioRxiv. doi:10.1101/027607:027607.

[9] Peterson D, Bonham KS, Rowland S, Pattanayak CW, Klepac-Ceraj V, Deoni SCL, D’Sa V, Bruchhage M, Volpe A, Beauchemin J, Wallace C, Rogers J, Cano R, Fernandes J, Walsh E, Rhodes B, Huentelman M, Lewis C, De Both MD, Naymik MA, Carnell S, Jansen E, Sadler JR, Thapaliya G, Bonham K, LeBourgeois M, Mueller HG, Wang J-L, Zhu C, Chen Y, Braun J. 2021. Comparative analysis of 16S rRNA gene and metagenome sequencing in pediatric gut microbiomes. Front Microbiol 12:670336. doi:10.3389/fmicb.2021.670336. 34335499 PMC8320171

[10] Bonk F, Popp D, Harms H, Centler F. 2018. PCR-based quantification of taxa-specific abundances in microbial communities: quantifying and avoiding common pitfalls. J Microbiol Methods 153:139–147. doi:10.1016/j.mimet.2018.09.015. 30267718

